# Supplementary material for: Farey tree locking of terahertz quantum cascade laser frequency combs
Source: Light Sci Appl. 2025 Mar 31;14:147. doi: 10.1038/s41377-025-01819-9 (PMC11958679; doi:10.1038/s41377-025-01819-9)
Supplement: Supplementary file 1 — Supplementary Information [file 41377_2025_1819_MOESM1_ESM.pdf]

Supplementary Information for

Farey tree locking of terahertz quantum cascade laser frequency combs

Guibin Liu[1,2], Xuhong Ma[1,2], Kang Zhou[1,3], Binbin Liu[1,2], Lulu Zheng[1,2], Xianglong Bi[1,2], Shumin Wu[1,2], Yanming Lu[1,2], Ziping Li[1], Wenjian Wan[1], Zhenzhen Zhang[1], Junsong Peng[4], Ya Zhang[5], Heping Zeng[3,4★], and Hua Li[1,2★]

[1]*State Key Laboratory of Materials for Integrated Circuits and Key Laboratory of Terahertz Solid State Technology, Shanghai Institute of Microsystem and Information Technology, Chinese Academy of Sciences, 865 Changning Road, Shanghai 200050, China.*

[2]*Center of Materials Science and Optoelectronics Engineering, University of Chinese Academy of Sciences, Beijing 100049, China.*

[3]*Chongqing Key Laboratory of Precision Optics, Chongqing Institute of East China Normal University, Chongqing 401120, China.*

[4]*State Key Laboratory of Precision Spectroscopy, East China Normal University, Shanghai 200241, China.*

[5]*Institute of Engineering, Tokyo University of Agriculture and Technology, Koganei-shi 184-8588 Tokyo, Japan*

★*Corresponding author. E-mail: hpzeng@phy.ecnu.edu.cn; hua.li@mail.sim.ac.cn.*

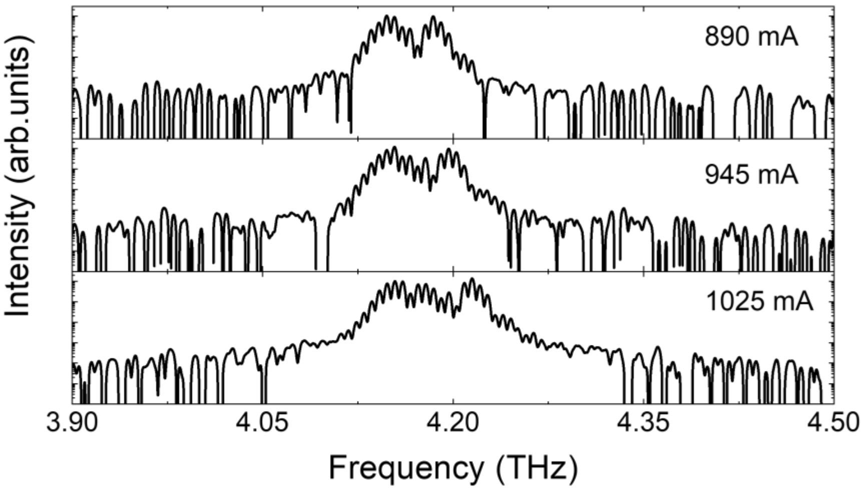

Fig. S1. Emission spectra of the free-running THz QCL measured at 890, 945, and 1025 mA (from top to bottom panels). The spectra were recorded using a Fourier transform infrared spectrometer (Bruker Vertex 80v) with a spectral resolution of 0.08 cm^{-1}.

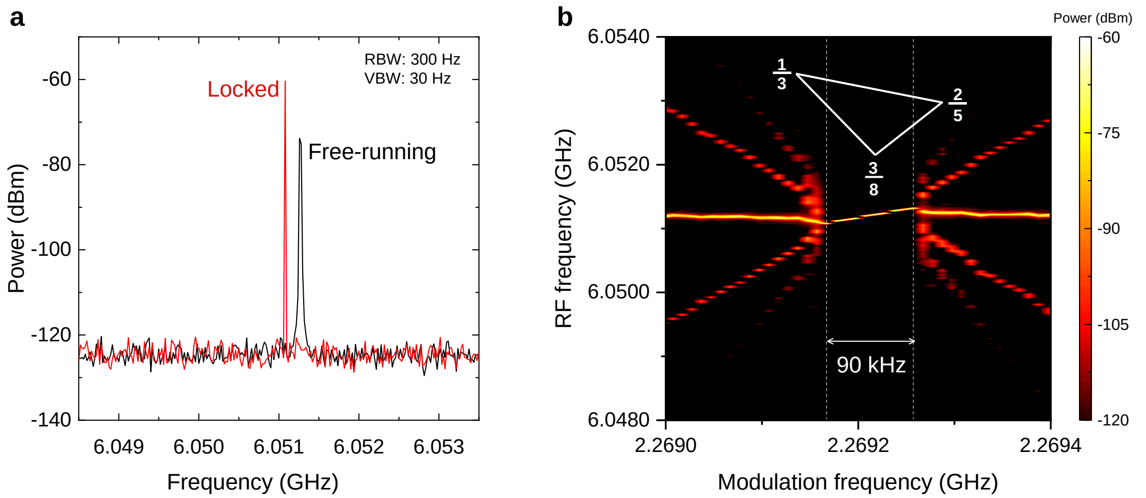

Fig. S2. Predicted Farey tree locking state with a Farey fraction 3/8. (**a**) Intermode beatnote measured in free-running (black) and Farey tree locking (red) state. Under the Farey tree locking condition, the intermode beatnote signal is significantly narrowed and the signal-to-noise ratio is improved by 12 dB compared with that measured in free-running mode. (**b**) Intermode beatnote map of the THz QCL comb as a function of modulation frequency (f_{mod}) measured near the winding number of 3/8. The step size of f_{mod} is 10 kHz. It shows a clear transition from an unstable region to the Farey tree locking region. The locking bandwidth is 90 kHz.

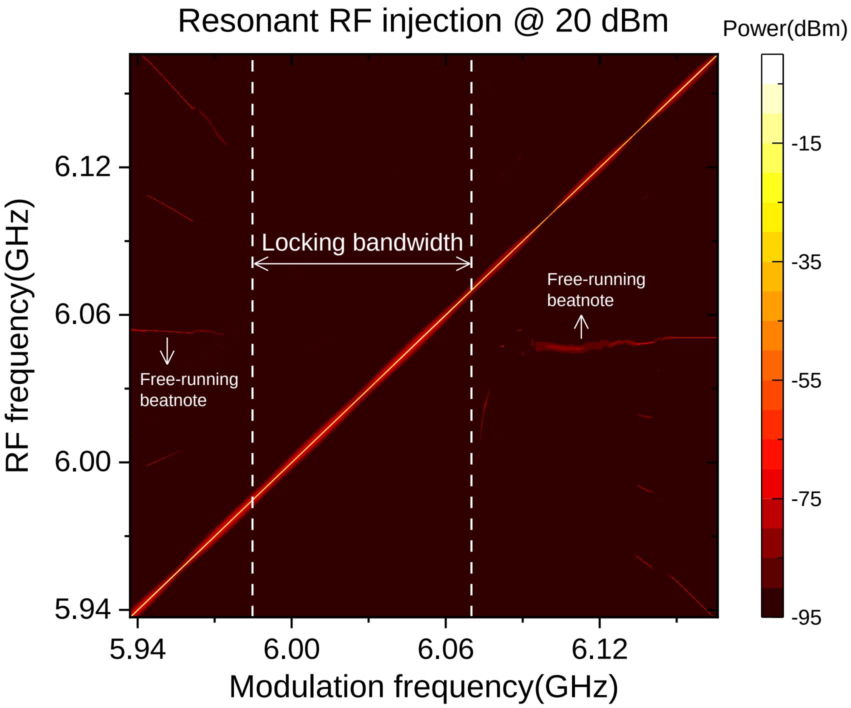

Fig. S3. Intermode beatnote map under resonant RF injection condition measured with an injection RF power of 20 dBm. The step size of the modulation frequency is 100 kHz. The free-running intermode beatnote frequency of the comb laser is around 6.05 GHz. For the measurement, the laser is stabilized at a heat sink temperature of 10 K.

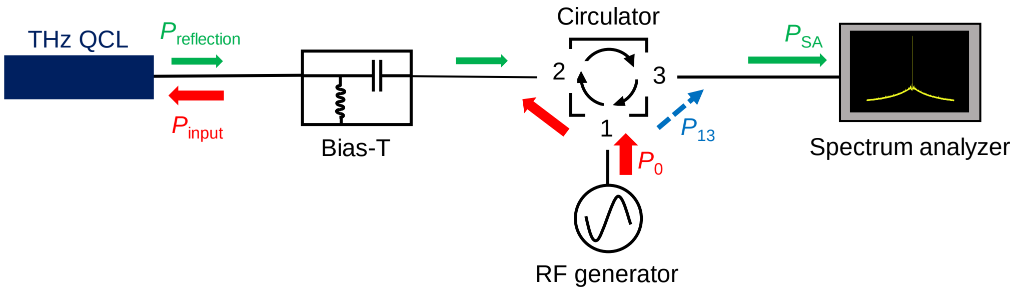

Fig. S4. Experimental setup for the measurement of the reflected driving RF power resulting from the impedance mismatch. Note that for the RF reflection measurement, the THz QCL is switched off without the DC electrical pumping. The red and green arrows schematically show the routes for the input and reflected RF signal, respectively. The blue dashed arrow indicates the signal leakage from port 1 to port 3 of the circulator due to its imperfect isolation.

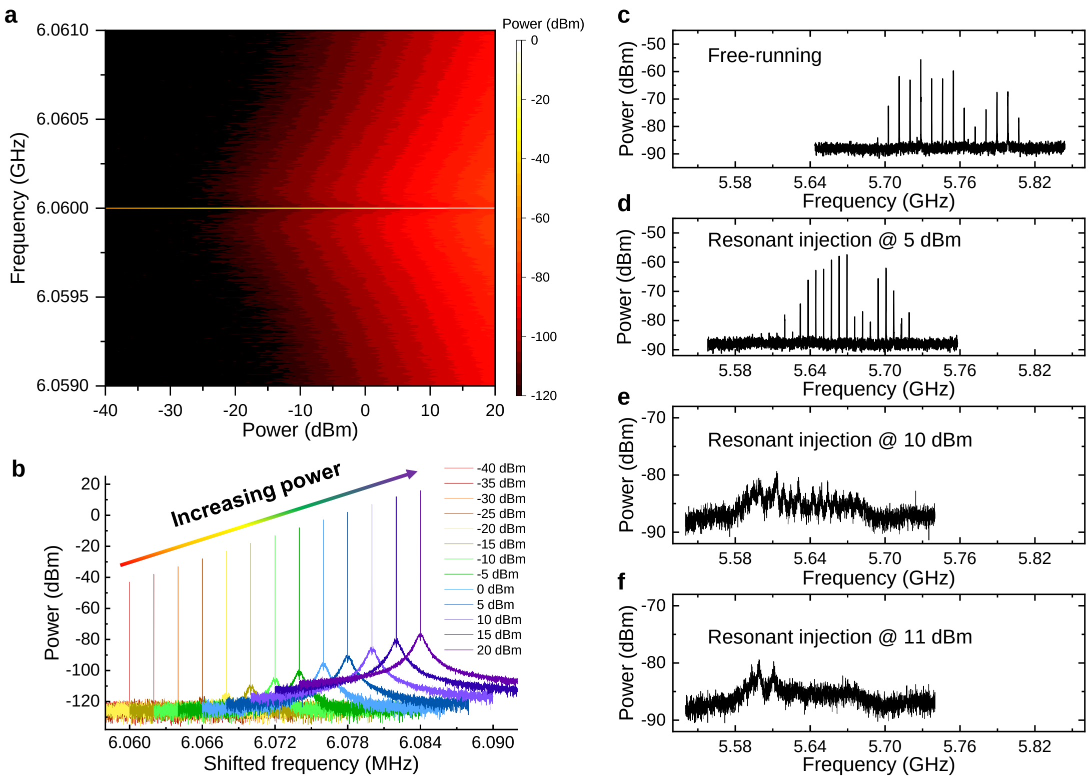

Fig. S5. (a) Map of the RF spectra of the RF synthesizer used in this work (Rohde & Schwarz SMA100B) by changing the RF power. The frequency of the RF synthesizer is fixed at 6.06 GHz, and the power step is 1 dBm. For the measurement, the RBW and VBW are set to 300 Hz and 30 Hz, respectively. **(b)** Cuts of (a) recorded at different RF power values. To make a clear comparison, the frequency is shifted. For the leftmost spectrum (-40 dBm), the frequency shift is 0. Afterwards, a 2-MHz frequency shift is applied to all other spectra in sequence. **(c)**, **(d)**, **(e),** and **(f)** show the measured dual-comb spectra recorded in free-running mode, resonant RF injection with a power of 5 dBm, resonant RF injection with a power of 10 dBm, and resonant RF injection with a power of 11 dBm, respectively. Due to the large phase noise introduced by the RF synthesizer at a high RF power of 11 dBm, the line-resolved dual-comb spectrum cannot be observed.

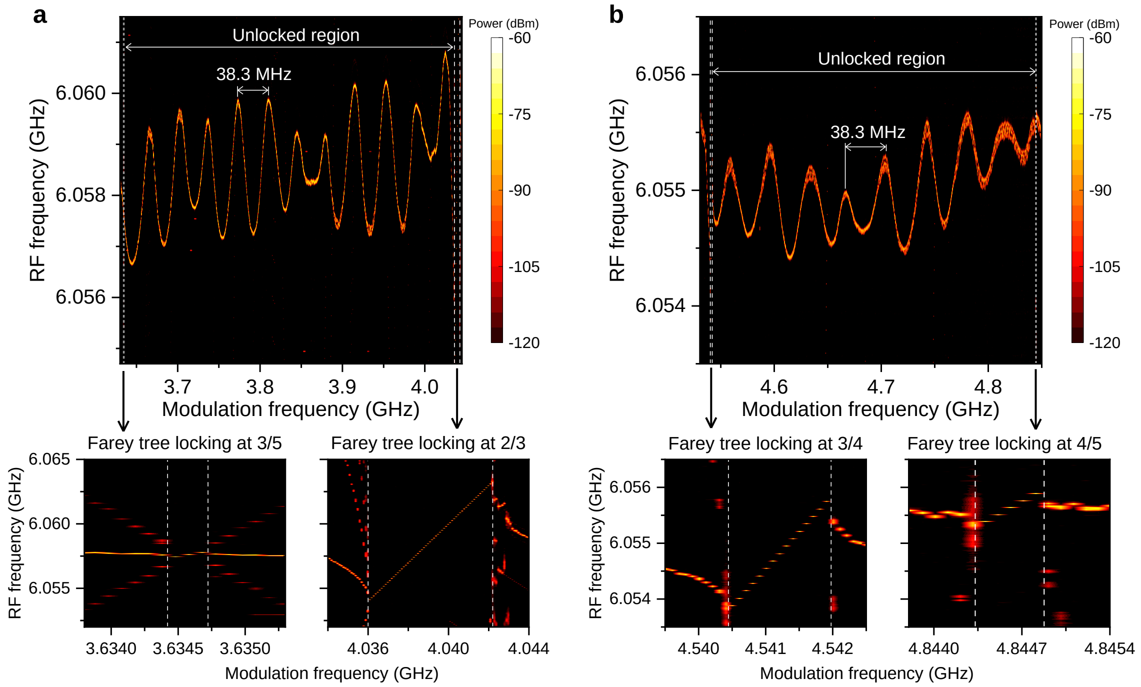

Fig. S6. Oscillation of repetition frequency with modulation frequency. (a) Repetition frequency map measured in the unlocked region between two Farey tree locking states with Farey fractions of 3/5 and 2/3. **(b)** Repetition frequency map measured in the unlocked region between two Farey tree locking states with Farey fractions of 3/4 and 4/5. The modulation power from the RF generator is fixed at 30 dBm, and the tuning step size is 100 kHz. At the bottom of the figure, four enlarged repetition frequency maps corresponding to the Farey tree locking at 3/5, 2/3, 3/4, and 4/5 are displayed from left to right panels.

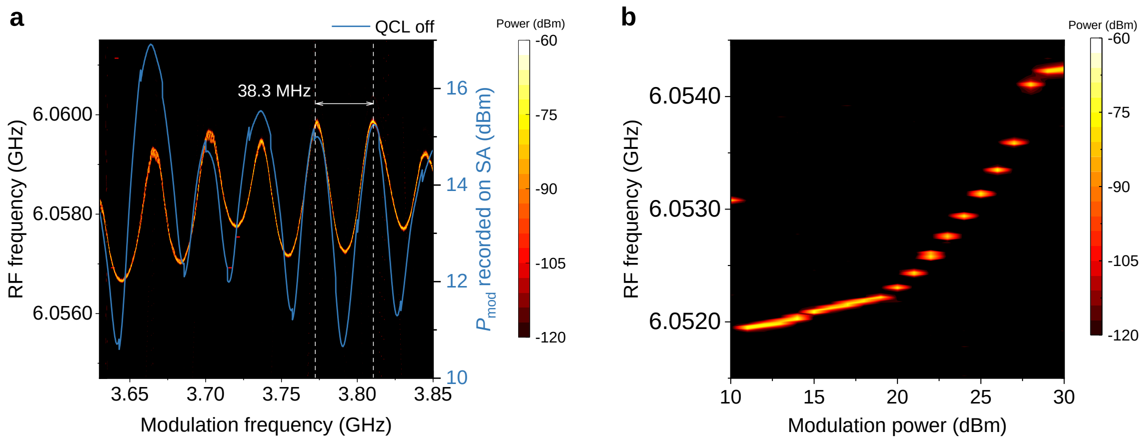

Fig. S7. (a) Periodic oscillation of intermode beatnote frequency and injected RF power with the change of modulation frequency. The orange curve shows the measured intermode beatnote frequency as a function of modulation frequency. The blue line depicts the modulation power (P_{mod}) recorded on the spectrum analyzer (SA) as a function of modulation frequency. The measurement of the blue line is based on the experimental setup in Fig. 1a of the main paper when the QCL is switched off. It can be seen that the two curves show similar oscillating behaviors with the modulation frequency and the period is 38.3 MHz. (**b**) Intermode beatnote map as the modulation power is varied. In this measurement, the modulation frequency is set to 3.7825 GHz and its power is greater than 10 dBm. We can see that f_{rep} monotonically increases with an increase in modulation power (step size of 1 dBm). An increase of 1.93 MHz in repetition frequency is observed as the modulation power increases from 20 to 30 dBm.

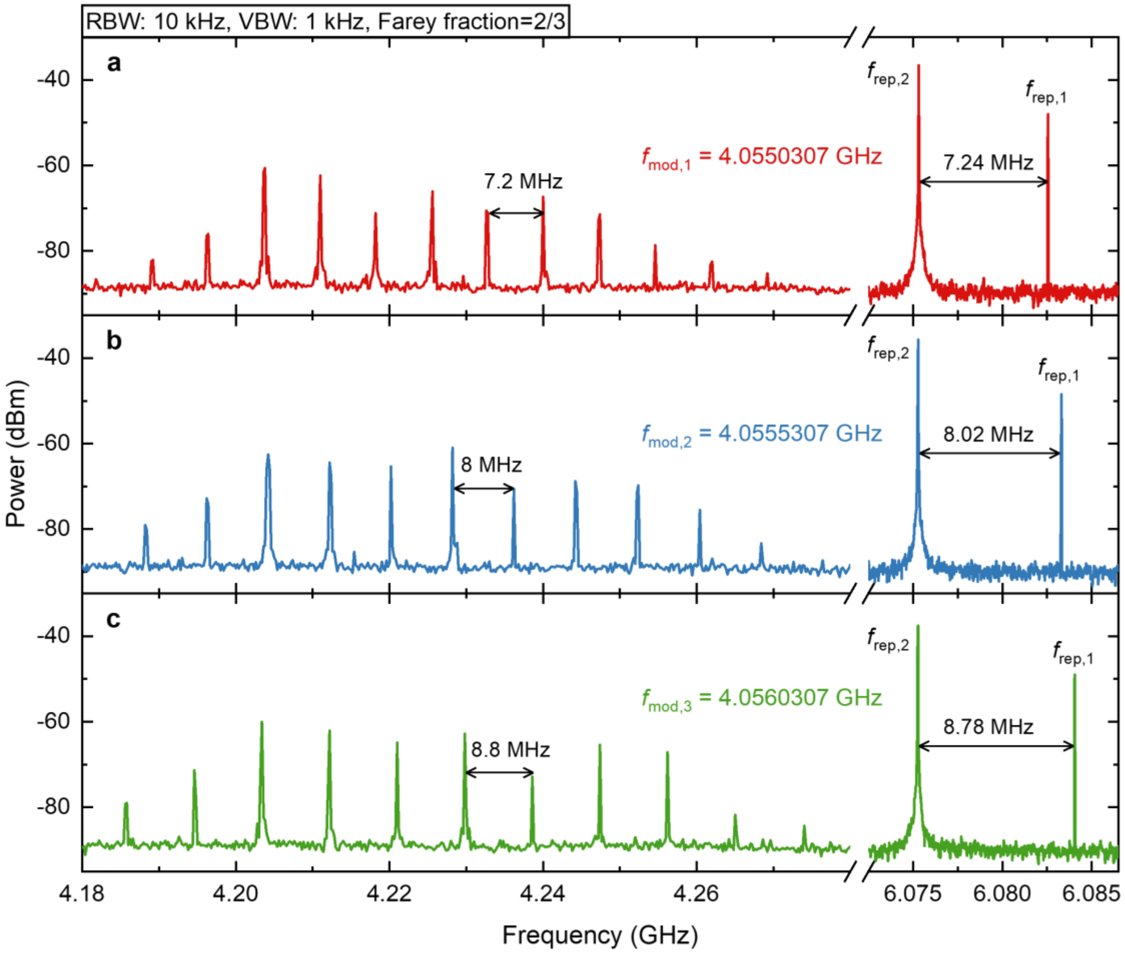

Fig. S8. Dual-comb spectra with tunable repetition frequencies. In the experiment, a Farey tree locking with a Farey fraction of 2/3 is implemented on Comb 1 to lock its repetition frequency ($f_{rep,1}$); while, Comb 2 is operated in free-running mode. (**a**) Dual-comb spectrum measured with $f_{mod,1}$=4.0550307 GHz, resulting in a repetition frequency difference (Δf_{rep}) of 7.24 MHz which is equal to the dual-comb line spacing. (**b**) Dual-comb spectrum measured with $f_{mod,2}$=4.0555307 GHz ($f_{mod,2}$=$f_{mod,1}$+0.5 MHz). The frequency tuning in modulation frequency results in an increase of dual-comb line spacing compared to the results shown in (a). (**c**) Dual-comb spectrum measured with $f_{mod,3}$=4.0560307 GHz ($f_{mod,3}$=$f_{mod,2}$+0.5 MHz). It is expected that the dual-comb line spacing is accordingly tuned as the modulation frequency is changed.

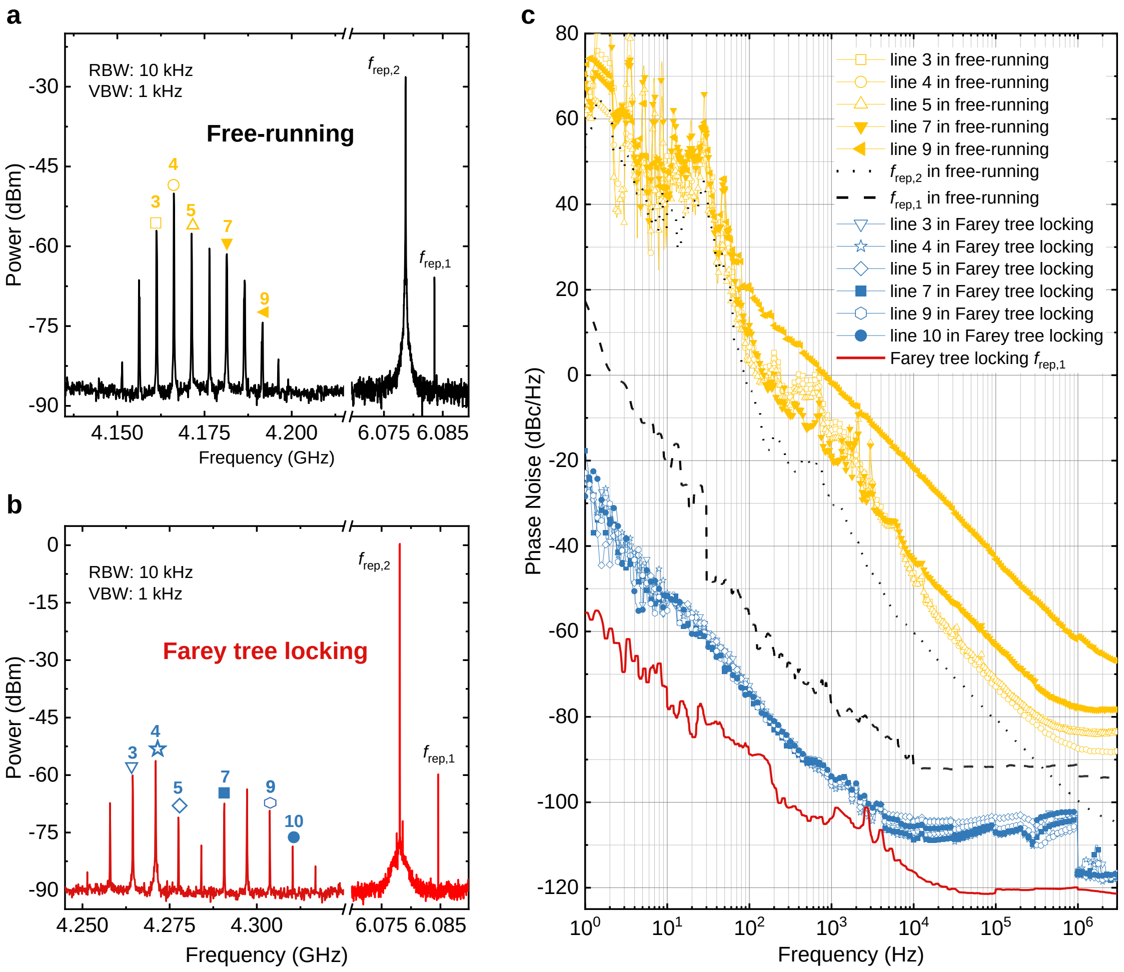

Fig. S9. (**a**) Dual-comb and intermode beatnote spectra recorded in free-running mode, which is the same as Fig. 7b of the main paper. The dual-comb lines are labeled from left to right as the index from 1 to 10, with lines 3, 4, 5, 7, and 9 specifically marked by different scatters. (**b**) Dual-comb and intermode beatnote spectra recorded under the Farey tree locking condition which is the same as Fig. 7c of the main paper. The dual-comb lines 3, 4, 5, 7, 9, and 10 are specifically marked using different scatters. (**c**) Phase noise spectra of the free-running dual-comb lines 3, 4, 5, 7, and 9 marked in (a) and the locked dual-comb lines 3, 4, 5, 7, 9, and 10 marked in (b). Phase noise spectra of dual-comb lines with the Farey tree locking exhibit consistent low phase noise performance. The offset frequency range is set from 1 Hz to 3 MHz (with the function of “low-pass filter” switched on and a cutoff frequency of 10 MHz). As a reference, the phase noise spectra of the free-running $f_{rep,1}$ (dotted line), free-running $f_{rep,2}$ (dashed line), and locked $f_{rep,1}$ (solid line) are also plotted. Note that we display every tenth point when plotting this figure to enhance the clarity of the date.

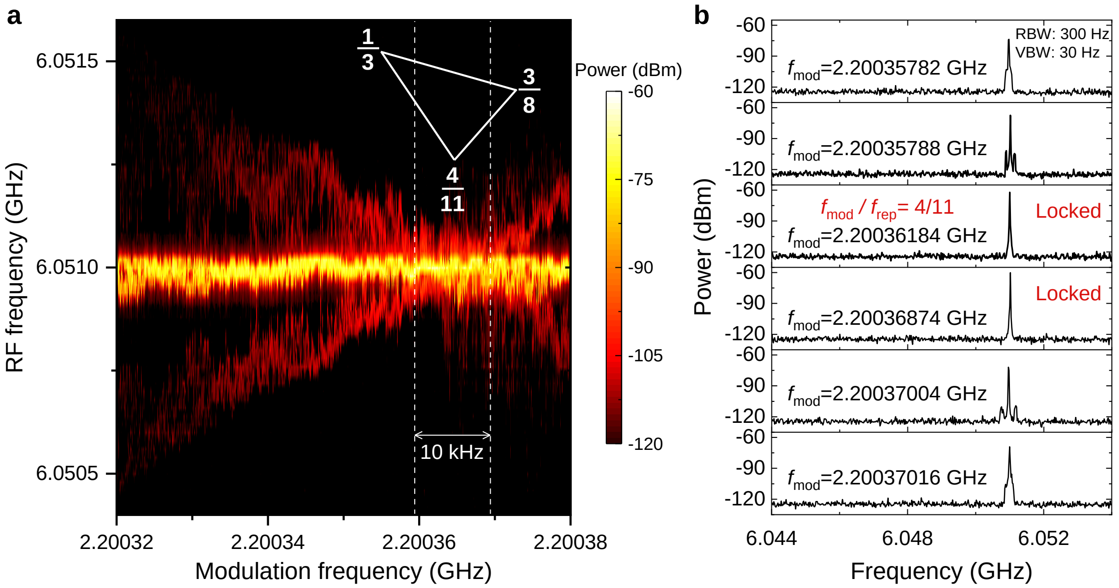

Fig. S10. Devil's staircase 4/11 fractal structure revealed by improving the frequency tuning accuracy. (**a**) Intermode beatnote map around winding number of 4/11. The modulation frequency is scanned step by step with a 20 Hz step size between the Farey fractions 1/3 and 3/8. The two vertical white dashed lines indicate the Farey tree locking bandwidth of 10 kHz around the winding number of 4/11. (**b**) Typical intermode beatnote spectra showing the evolution from free-running to Farey tree locking and finally back to free-running states at the Farey fraction 4/11.

Table S1: List of the measured power values and reflection ratios, e.g., power of RF generator (P_0), input power of the THz QCL device (P_{input}), leakage power (P_{13}), power recorded on the spectrum analyzer (P_{SA}), reflected power by the THz QCL device ($P_{reflection}$), and reflection ratio, by tuning the power of the RF generator (P_0) from -20 to 20 dBm with a step of 5 dBm. The operation frequency for this measurement is set as 6 GHz which is close to the intermode beatnote frequency of the comb laser. Note that the RF transmission attenuation introduced by the circulator and bias-T, approximately 6 dB, as well as the circulator's isolation of 23 dB, are taken into account.

Power of RF generator, P_0 (dBm)	Input power, P_{input} (dBm)	Leakage power, P_{13} (dBm)	Power recorded on spectrum analyzer, P_{SA} (dBm)	Reflected RF driving power, $P_{reflection}$ (dBm)	Reflection ratio (dB)
-20	-26.48	-43	-37.87	-33.46	-6.98
-15	-21.47	-38	-32.88	-28.47	-7
-10	-16.47	-33	-27.87	-23.46	-6.99
-5	-11.46	-28	-22.88	-18.48	-7.02
0	-6.45	-23	-17.87	-13.45	-7
5	-1.23	-18	-12.74	-8.28	-7.05
10	3.77	-13	-7.74	-3.28	-7.05
15	8.76	-8	-2.75	1.71	-7.05
20	12.74	-3	1.43	5.49	-7.25

Table S2: Farey tree locking plateaus extracted from Fig. 6a of the main paper.

Farey fraction	$f_{mod,left}$ (GHz)	$f_{mod,right}$ (GHz)	Plateau width (MHz)
1/3	2.016880000	2.016980000	0.1
3/8	2.269167250	2.269257250	0.09
2/5	2.419920000	2.421120000	1.2
1/2	3.025976773	3.034093656	8.1
4/7	3.457748600	3.457816500	0.0679
3/5	3.631600000	3.631800000	0.2
2/3	4.033100000	4.038600000	5.5
3/4	4.540400000	4.541900000	1.5
4/5	4.844400000	4.844900000	0. 5
5/6	5.046774000	5.047004000	0. 23
